# Enhanced subfibular ossicle diagnosis: CT-MRI integration of morphology and ligament attachments

**DOI:** 10.1186/s13244-025-02138-8

**Published:** 2025-11-12

**Authors:** Nan Xu, Peng Sun, Jun Zhang, Ke Tian, Qian Gao, Xiang-Sheng Li

**Affiliations:** 1 Department of Radiology, Air Force Medical Center, Air Force Medical University, Beijing, China; 2 Department of Radiology, Sir Run Run Hospital, Nanjing Medical University, Nanjing, China

**Keywords:** Subfibular ossicle, Os subfibulare, Nonunited avulsion fracture, Computed tomography, Magnetic resonance imaging

## Abstract

**Purpose:**

To assess the diagnostic value of CT and MRI in distinguishing nonunited avulsion fracture (NAF) of the lateral malleolus from os subfibulare (OSF).

**Materials and methods:**

In this retrospective study, 114 subfibular ossicles (SFOs) in 108 patients were evaluated by CT and MRI for shape, margin, size, CT attenuation, spatial orientation, and anatomical relations. Surgical, arthroscopic, and follow-up findings served as reference standards. Logistic regression and receiver operating characteristic (ROC) analyses assessed diagnostic performance.

**Results:**

NAFs were associated with higher incidences of lateral ankle pain (92.3% vs. 71.4%, *p* = 0.003) and instability (29.2% vs. 10.2%, *p* = 0.014) than OSF. CT showed that NAF had a more irregular shape, rougher margins, higher attenuation, and anteroposterior orientation compared to OSF (all *p* < 0.05). MRI revealed ligamentous attachment predominated in NAF (72.3%), while OSF mostly showed discontinuity or apposition (91.8%, *p* < 0.001). The combined CT model achieved an AUC of 0.782, accuracy of 74.3%, sensitivity of 91.9%, and specificity of 56.8%. MRI-based ligamentous attachment MRI strict criterion yielded an AUC 0.821, with a sensitivity of 72.3% and a specificity of 91.8%, while the MRI inclusive criterion was 0.752, 95.4% and 55.1%. Ossicle size correlated with symptom severity (*p* < 0.001).

**Conclusion:**

MRI is highly effective for differentiating NAF from OSF by directly visualizing SFO-ligament attachments, while CT provides complementary morphological detail. The combined use of CT and MRI delivers robust diagnostic performance, supporting clinical decision-making in lateral malleolar lesions.

**Critical relevance statement:**

CT and MRI can distinguish a nonunited avulsion fracture from os subfibulare. Especially, MRI can visually display the relationship between SFO and the lateral collateral ligament.

**Key Points:**

- Distinguishing chronic lateral malleolus avulsion fractures from congenital accessory bones remains diagnostically challenging.
- MRI directly visualizing ligament attachment to the bone fragment best differentiates fractures from accessory bones.
- Combining MRI and CT findings provides comprehensive evidence for clinical management decisions.

**Graphical Abstract:**

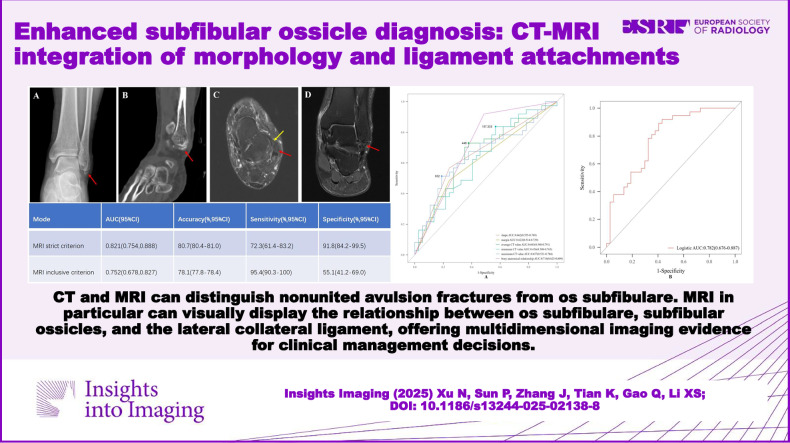

## Introduction

The term subfibular ossicle (SFO) collectively describes osseous fragments at the fibular tip, encompassing both nonunited avulsion fracture (NAF) and the accessory bone os subfibulare (OSF) [1–3]. While OSF is uncommon (prevalence 0.42–1.00%) and typically unilateral [4–6], it may confer biomechanical advantages, potentially stabilizing the forefoot and enhancing plantar flexion [7]. Usually asymptomatic and incidentally detected on radiographs obtained for ankle sprains or other injuries, OSF can become symptomatic due to trauma or overuse, leading to pain or degenerative changes [8, 9]. In contrast, NAF are more frequently associated with lateral ankle pain, instability, and an elevated risk of secondary osteoarthritis, particularly as a contributor to lateral ankle instability [3, 10–14]. Surgical intervention is often indicated for symptomatic SFOs refractory to conservative management, though the optimal approach remains debated and varies by SFO type and size [1, 13, 15–17]. Therefore, accurate differentiation of SFO types and comprehensive morphological assessment are essential to guide appropriate clinical management.

Anatomical studies have confirmed that OSF is a congenital variant devoid of direct ligamentous or capsular attachments [7]. Surgical and arthroscopic findings can serve as reference standards for distinguishing OSF from NAF [9, 18]. Histologically, OSF exhibits a fibrocartilaginous surface without ligamentous insertions at either pole [6]. In contrast, NAF maintains ligamentous continuity, primarily involving the anterior talofibular ligament (ATFL), and less frequently the posterior talofibular ligament (PTFL) or calcaneofibular ligament (CFL) [13, 19]. Histology of NAF reveals non-specific osseous fragments surrounded by reactive synovium with vascularized margins, without endochondral ossification [19]. Acute avulsion fractures are usually easy to identify on imaging-they appear as irregular, sharp-edged bone fragments near the fibular tip; however, NAF and OSF may exhibit similar features, with smooth edges and rounded shapes, making them hard to distinguish on X-rays and even CT [1, 11, 20]. MRI provides multiplanar, multisequence assessments of the relationship between ATFL and SFO, with case reports, case series, and small pediatric sample studies suggesting that ligamentous attachment to the SFO favors NAF, while absence of attachment suggests OSF [6, 14, 18, 21–23]. MRI also assesses complications such as synovitis and impingement in symptomatic NAF and helps predict poor response to conservative therapy, thereby guiding management [14, 17, 21].

Currently, studies primarily focus on the clinical management of SFO, lacking systematic large-sample imaging studies. Therefore, this study aims to evaluate the diagnostic value of CT and MRI, particularly the ligament attachment relationships. We indicated that MRI can visually display the anatomical relationship between the SFO and the lateral collateral ligaments of the ankle. This study not only refines imaging protocols but also provides a foundation for future studies on subfibular pathology management and optimal imaging strategies.

## Materials and methods

### Participants

This retrospective cohort study enrolled patients with SFO identified incidentally or during symptomatic evaluation at the Air Force Medical Center from March 2013 to March 2025. The study was approved by the Medical Ethics Committee (2025-11-PJ01), and patient informed consent was waived. Inclusion criteria: (1) Imaging confirmation of discrete osseous fragments at the distal lateral malleolus; (2) SFO diagnosis confirmed surgically or arthroscopically; (3) SFO confirmed by follow-up imaging (after excluding acute avulsion fractures at initial presentation, follow-up demonstrated unchanged morphology) [23–25]. Exclusion criteria: (1) Acute lateral malleolar avulsion fracture due to; (2) Prior ankle surgery; (3) Ankle osteoarthritis; (4) Concomitant distal fibular fracture; (5) Incomplete imaging data.

### CT protocol

Non-contrast ankle CT was performed on a GE LightSpeed 16-slice scanner with patients supine, feet-first. Acquisition parameters: 120 kV tube voltage, 200 mA tube current, 5-mm slice thickness with 5-mm intervals for standard axial sections, and 1.25-mm slice thickness with 1-mm intervals for thin-section reconstructions.

### MRI protocol

Ankle joint scans were performed using the GE Discovery MR750 3.0-T MRI scanner. The sequences and parameters are as follows: Sagittal plane, PDWI-FS (TR 2205 ms, TE 37.9 ms), T1WI (TR 484 ms, TE 11.2 ms); Coronal plane, PDWI-FS (TR 2515 ms, TE 44.3 ms); Axial plane, PDWI-FS (TR 2738 ms, TE 50.4 ms), T1WI (TR 496 ms, TE 15.7 ms); general parameters, matrix 512 × 512, 4-mm slice thickness with 0.4-mm slice gap. It should be noted that the 4-mm slice thickness may result in partial volume effects, potentially limiting the detailed assessment of small ossicles and their associated ligamentous structures. This limitation is further discussed in the “Discussion” section.

### Imaging analysis [3, 14, 17, 21]

CT evaluation encompassed seven metrics: (1) shape, regular (round or oval) or irregular; (2) margin, smooth (cortical continuity with homogeneous density) or rough (cortical discontinuity, defect, or heterogeneity in any plane); (3) size, maximum longitudinal diameter across on coronal, sagittal, and axial planes, categorized as < 5 mm (small), 5–10 mm (medium), > 10 mm (large); (4) region of interest (ROI) delineation of CT value: Two radiologists independently outlined the ROI, selecting the largest cross-section of the bone on axial, sagittal, and coronal planes, using the clearly visualized hyperdense cortical bone as the boundary. Post-processing software automatically calculated the mean, minimum, and maximum CT values. Each radiologist performed three measurements and averaged the results, and the final value was obtained by averaging the mean values from both radiologists; (5) location (relative to the fibular tip), anteromedial (ATFL region), posteromedial (PTFL region), and inferior (CFL region); (6) long-axis orientation, anteroposterior, transverse and craniocaudal; (7) bony anatomical relationship with lateral malleolus, compatible or noncompatible.

**Fig. 1:**
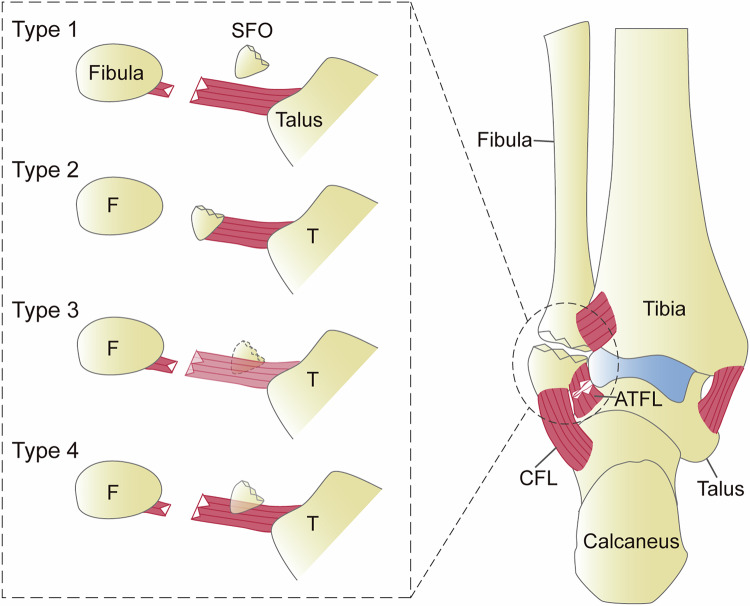
MRI schematic of the ligament-SFO relationship. Type 1 (discontinuous), the ligament shows no association with the SFO; Type 2 (contiguous), the ligament directly attaches to the SFO; Type 3 (apposed), the SFO is located within the course of the ligament but is visualized in adjacent imaging planes rather than the same plane; Type 4 (apposed), the SFO lies within the course of the ligament without direct attachment

MRI assessment included two parameters: Relationship between the SFO and the lateral collateral ligament, classified as contiguous, discontinuous and apposed; predictors of conservative treatment failure, interposition of fluid-signal intensity at lateral malleolus-SFO interface on PDWI-FS and SFO bone marrow edema. Three distinct ligament-SFO relationships were defined: (1) “discontinuous,” complete ligament visualization along its course or SFO situated outside the trajectory of the ligament; (2) “contiguous,” unequivocal ligament origin attachment to the SFO; (3) “apposed,” SFO positioned within the course of the ligament but without direct attachment [6, 14, 17, 18, 22, 26]. Based on the MRI strict criterion, the “contiguous” configuration was classified as NAF, whereas both “discontinuous” and “apposed” were categorized as OSF. In contrast, under the MRI inclusive criterion, only the “discontinuous” state was designated as OSF (Fig. 1).

Blind image interpretation was independently performed by two musculoskeletal radiologists, each with > 10 years of experience. Both were blinded to clinical data and final outcomes. In cases of disagreement, a final assessment was provided by a senior physician with > 20 years of experience.

### Reference criteria for diagnosing OSF and NAF

The diagnosis of SFO was confirmed through surgery, arthroscopy, and follow-up evaluations. Surgical methods included bone resection, internal bone fixation, ligament reconstruction, and ligament repair.

#### Consistency analysis

Interrater agreement between two observers was assessed using the intraclass correlation coefficient (ICC) for qualitative variables (shape, margin, location, orientation of the longest axis, bony and ligament anatomical relationships) and Cohen’s Kappa (*κ*) for quantitative variables (size, average/minimum/maximum CT values), calculated with the irr and vcd packages, respectively. Statistical significance was set at two-sided *p* < 0.05. Agreement levels were defined as moderate (0.41–0.60), good (0.61–0.80), and excellent (0.81–1.00).

### Statistical analyses

All analyses were performed using R (v4.2.2). Normally distributed continuous variables were compared using independent *t*-tests (two groups) or ANOVA (≥ 3 groups), expressed as mean ± SD; nonparametric data were reported as median. Categorical variables used Pearson’s χ² or Fisher’s exact tests. Post hoc pairwise comparisons employed Bonferroni correction. Univariate logistic regression identified significant CT parameters; multivariate regression derived a combined diagnostic model incorporating shape, margin, size, average/minimum/maximum CT values, location, long-axis orientation, and bony relationship to the lateral malleolus. ROC analysis evaluated performance. Two-sided *p* < 0.05 defined significance.

## Results

### Patient clinical characteristics

**Fig. 2:**
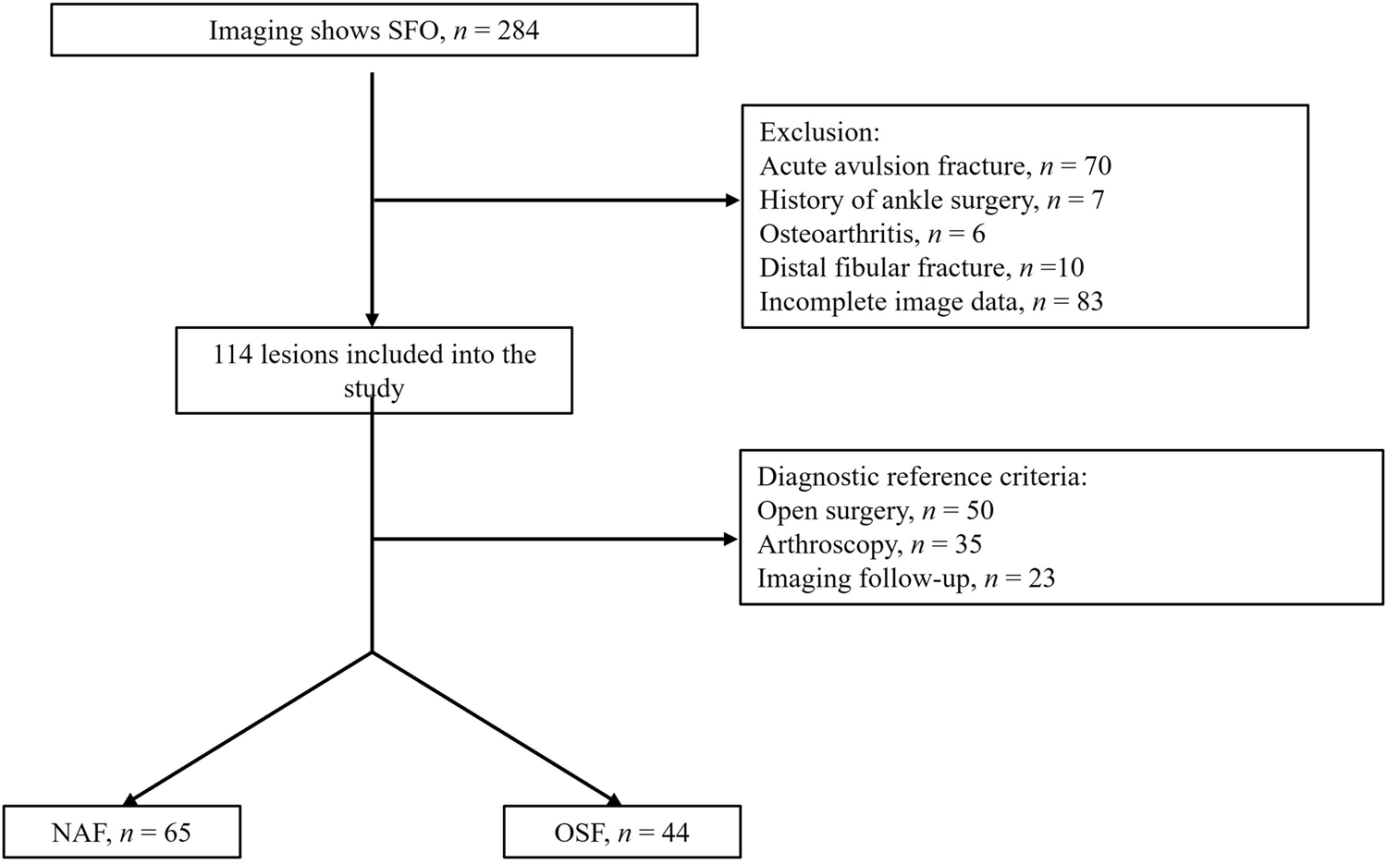
Study inclusion flowchart

A total of 108 patients were included (74 males, 34 females; mean age 27.3 years, range 15–50), revealing 114 SFOs (52 on the left side and 62 right). Diagnoses included 65 NAF (62 unilateral, 1 bilateral), 49 OSF (40 unilateral, 4 bilateral), and one special patient with NAF and OSF on both sides respectively (Fig. 2). Reasons for presentation included lateral malleolus skin infection (*n* = 1), chronic ankle discomfort (*n* = 77, 55 with ankle sprain history, 3 with fall history, and 19 without significant trauma), incidental detection (*n* = 26, 21 during bilateral examinations, 5 after traffic accidents), calcaneal fracture (*n* = 2), medial malleolus fracture (*n* = 1), and suspected osteoarthritis (*n* = 1). Clinical manifestations were lateral ankle pain with instability (*n* = 24, 21.1%), isolated lateral ankle pain (*n* = 71, 62.3%), and asymptomatic (*n* = 19, 16.7%). Male predominance was observed in both groups. Compared with the OSF group, the NAF group demonstrated higher proportions of patients presenting with both lateral ankle pain with instability and isolated lateral ankle pain (Table 1).

**Table 1:** Summary of patient characteristics

	NAF *N* = 65 (57%)^a^	OSF *N* = 49 (43%)^a^	*t/Z/χ*^2^^b^	*p*-value^b^
Age (years)	27.7 ± 9.6	25.2 ± 7.0	1.635	0.102
Gender			0.812	0.368
Female	16 (24.6%)	18 (36.7%)		
Man	49 (75.4%)	31 (63.3%)		
Side			1.013	0.314
Left	27 (41.5%)	25 (51.0%)		
Right	38 (58.5%)	24 (49.0%)		
Instability			6.085	0.014
Negative	46 (70.8%)	44 (89.8%)		
Positive	19 (29.2%)	5 (10.2%)		
Pain			8.769	0.003
Negative	5 (7.7%)	14 (28.6%)		
Positive	60 (92.3%)	35 (71.4%)		

^a^ Median (Q1, Q3); *n* (%)

^b^ Wilcoxon–Mann–Whitney test; Pearson’s Chi-squared test

### Interobserver reproducibility

Due to the clear definitions established for each CT and MRI parameter, junior physicians were able to make clear determinations for most variables, resulting in very high agreement between the two modalities. Consequently, disagreements during image review were rare. Potential subjective ambiguities were noted only in the assessment of the margin and the relationship between the SFO and the lateral collateral ligament. Nevertheless, the results still demonstrated excellent consistency in these areas (Table S1).

### Comparison of CT between NAF and OSF and the diagnostic performance of CT

CT assessment of 114 SFOs showed: shape regular in 42 cases (36.8%) and irregular in 72 (63.2%); margins smooth in 45 cases (39.5%) and rough in 69 (60.5%); mean size 9.16 mm (range: 3.8–13.8 mm), subcategorized as large in 45 (39.5%), medium in 61 (53.5%), and small in 8 (7%); location anteromedial in 105 (92.1%), posteromedial in 2 (1.8%), and inferior in 7 (6.1%); long-axis orientation anteroposterior in 92 (80.7%), craniocaudal in 11 (9.6%), and transverse in 11 (9.6%); compatible bony anatomical relationship to the lateral malleolus in 47 cases (41.2%) and noncompatible in 67 (58.8%) (Table S2).

Intergroup comparisons showed that NAF had significantly higher rates of irregular shape, rough margin, and anteroposterior orientation than OSF, with higher mean, maximum, and minimum CT attenuation values (*p* < 0.05). Conversely, OSF was more often anatomically incompatible with the lateral malleolus. No significant differences were found in SFO size or positional distribution. Both groups were predominantly medium to large fragments located anteromedially (Table S2).

**Fig. 3:**
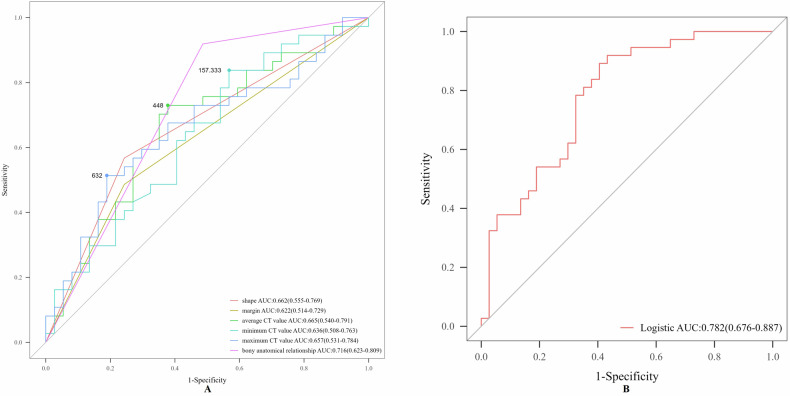
The ROC curve of CT for the differential diagnosis of NAF and OSF. **A** Single model. **B** Combined model

Shape, margin, average/minimum/maximum CT values, and bony anatomical relationships had diagnostic value in differentiating NAF from OSF (Table 2). In a multivariable logistic model combining these parameters, ROC analysis showed the highest overall accuracy and sensitivity, with moderate specificity (Fig. 3). Notably, the maximum CT value alone yielded the highest specificity (Table 2).

**Table 2:** The diagnostic performance of CT in differentiating NAF from OSF

Mode	AUC (95% CI)	Accuracy (%, 95% CI)	Sensitivity (%, 95% CI)	Specificity (%, 95% CI)
Shape	0.662 (0.555, 0.769)	66.2 (65.6–66.8)	56.8 (40.8–72.7)	75.7 (61.9–89.5)
Margin	0.622 (0.514, 0.729)	62.2 (61.5–62.8)	48.6 (32.5–64.8)	75.7 (61.9–89.5)
Avg CT	0.665 (0.540, 0.791)	67.6 (67.0–68.1)	73.0 (58.7–87.3)	62.2 (46.5–77.8)
Min CT	0.636 (0.508, 0.763)	63.5 (62.9–64.1)	83.8 (71.9–95.7)	43.2 (27.3–59.2)
Max CT	0.657 (0.531–0.784)	66.2 (65.6–66.8)	51.4 (35.2–67.5)	81.1 (68.5–93.7)
Baf	0.716 (0.623, 0.809)	71.6 (71.1–72.2)	91.9 (83.1–100)	51.4 (35.2–67.5)
Logistic	0.782 (0.676, 0.887)	74.3 (73.8–74.8)	91.9 (83.1–100)	56.8 (40.8–72.7)

*Avg CT* average CT value, *Min CT* minimum CT value, *Max CT* maximum CT value, *Baf* bony anatomical relationship with lateral malleolus

### Comparison of MRI between NAF and OSF and the diagnostic performance

MRI revealed fluid-signal interposition on PDWI-FS at the SFO-lateral malleolar space interface in 65.8% of cases (75/114), and SFO bone marrow edema in 22.8% (26/114) (Table 3). Regarding relationships to lateral malleolar ligaments, SFOs were contiguous in 51 cases (44.7%, including 1 connected to the CFL), apposed in 33 (28.9%), and discontinuous in 30 (26.3%) (Table 3).

**Table 3:** NAF versus OSF in MRI

Characteristic	NAF *n* = 65 (57%)^a^	OSF *n* = 49 (43%)^a^	*t/Z/χ*^2^^b^	*p*-value^b^
Ligament anatomical relationship			54.557	< 0.001
Apposed	15 (23.1%)	18 (36.7%)		
Contiguous	47 (72.3%)	4 (8.2%)		
Discontinuous	3 (4.6%)	27 (55.1%)		
MRI-interposition of fluid-signal intensity			0.182	0.670
Negative	21 (32.3%)	18 (36.2%)		
Positive	44 (67.7%)	31 (63.8%)		
MRI-bone marrow edema			3.145	0.076
Negative	46 (70.8%)	42 (85.7%)		
Positive	19 (29.2%)	7 (14.3%)		

^a^ *n* (%); Mean ± SD; Median (Q1, Q3)

^b^ Pearson’s Chi-squared test; Fisher’s exact test for count data; two sample *t*-test; Wilcoxon–Mann–Whitney test

In NAF, SFOs were predominantly ligament-connected, whereas in OSF, they were mainly discontinuous or apposed. Although fluid-signal interposition was more frequent overall, it did not differ significantly between groups (*p* > 0.05). Additionally, the incidence of SFO edema was low and also showed no significant intergroup difference (Table 3).

**Fig. 4:**
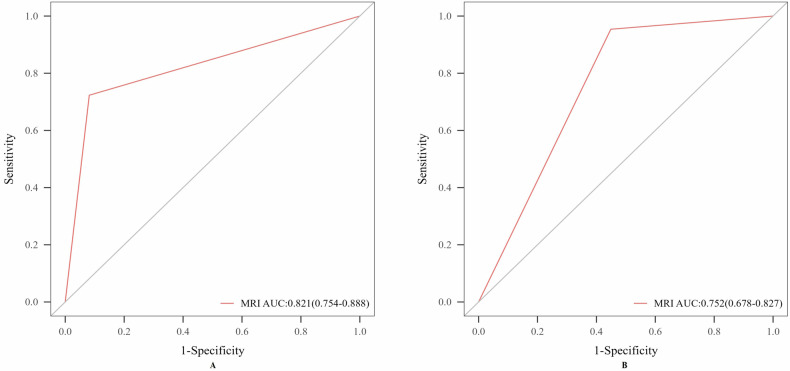
The ROC curve of MRI for the differential diagnosis of NAF and OSF. **A** MRI strict criterion. **B** MRI inclusive criterion. MRI strict criterion: SFO connected to the ligament is classified as NAF, while intersecting or not connected is classified as OSF; MRI inclusive criterion: SFO connected to or intersecting with the ligament is classified as NAF, while not connected is classified as OSF

**Fig. 5:**
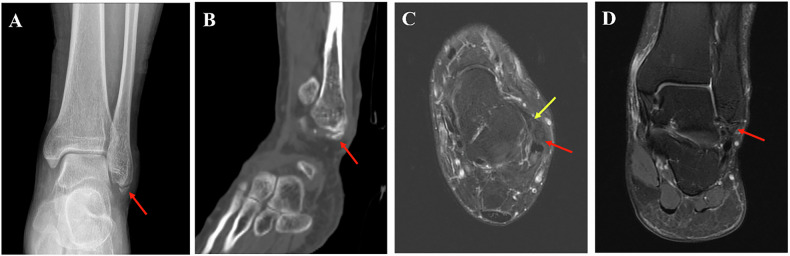
Imaging findings in a 41-year-old male NAF patient. **A** Ankle anteroposterior radiograph: the SFO (red arrow) exhibits a congruent relationship with the lateral malleolus. **B** Sagittal CT reconstruction (bone window): the SFO (red arrow) demonstrates an irregular contour, cortical irregularity, and an anteroposterior long-axis orientation. **C** Axial PDWI: the SFO (red arrow) is located anteromedial to the lateral malleolus and exhibits continuity with ATFL (yellow arrow). **D** Coronal PDWI: the SFO (red arrow) displays an absence of T2-hyperintense signal between it and the fibula, and the ossicle itself shows no evidence of associated marrow edema

**Fig. 6:**
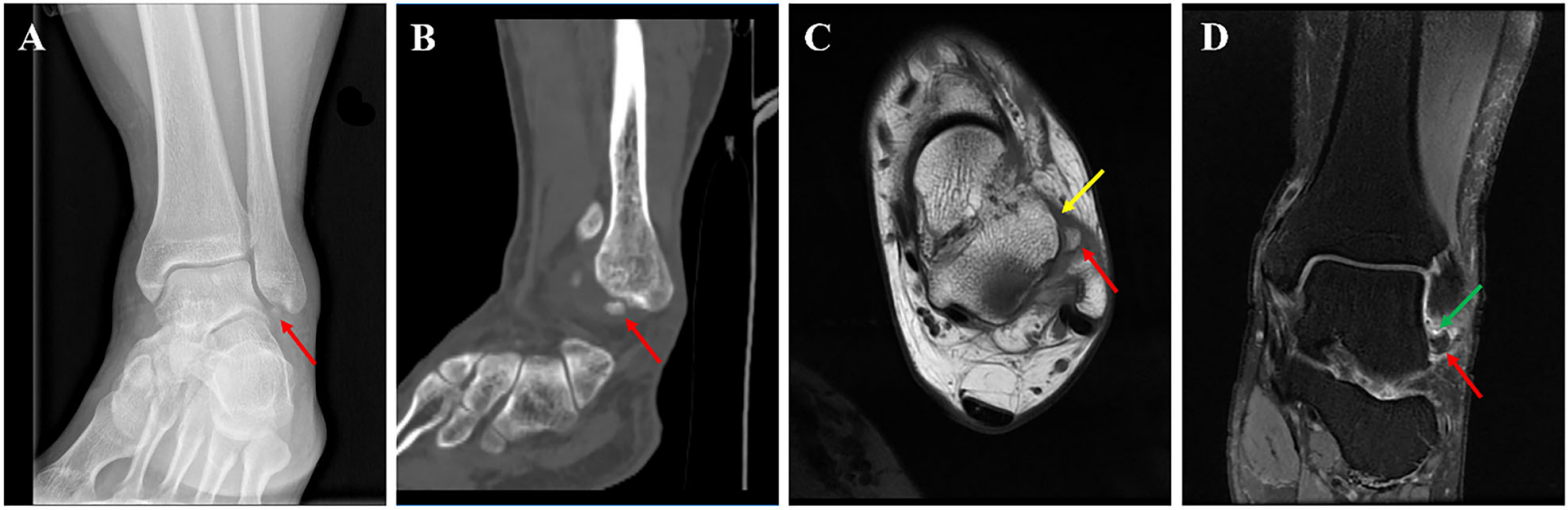
Imaging findings in a 29-year-old male OSF patient. **A** Ankle anteroposterior radiograph: the SFO (red arrow) demonstrates an incongruent relationship with the lateral malleolus. **B** Sagittal CT reconstruction (bone window): the SFO (red arrow) exhibits an irregular contour, cortical irregularity, and an anteroposterior long-axis orientation. **C** Axial T1WI: the SFO (red arrow) is located anteromedial to the lateral malleolus and contacts the ATFL (yellow arrow). **D** Coronal PDWI: intervening T2-hyperintense signal is present between the SFO (red arrow) and the fibula (green arrow), and the ossicle itself shows no evidence of associated marrow edema

MRI strict criterion defined a contiguous SFO-ligament relationship as NAF and discontinuous or apposed as OSF, demonstrating high accuracy and specificity but limited sensitivity. Conversely, MRI inclusive criterion classified solely no attachment as OSF, which significantly increased sensitivity (from 72.3 to 95.4%) while reducing specificity (Tables 4, 5 and Figs. 4–6).

**Table 4:** Comparison of MRI using strict and inclusive criteria for the differential diagnosis of SFO

Characteristic		Gold standard		
		NAF *n* = 65 (57%)	OSF *n* = 49 (43%)	*p*-value
MRI strict criterion	NAF	47 (72.3%)	4 (8.2%)	< 0.001
	OSF	18 (27.7%)	45 (91.8%)	
MRI inclusive criterion	NAF	62 (95.4%)	22 (44.9%)	< 0.001
	OSF	3 (4.6%)	27 (55.1%)	

MRI strict criterion: SFO connected to the ligament is classified as NAF, while intersecting or not connected is classified as OSF; MRI inclusive criterion: SFO connected to or intersecting with the ligament is classified as NAF, while not connected is classified as OSF

**Table 5:** The diagnostic performance of MRI using strict and inclusive criteria in differentiating NAF from OSF

Mode	AUC (95% CI)	Accuracy (%, 95% CI)	Sensitivity (%, 95% CI)	Specificity (%, 95% CI)
MRI strict criterion	0.821 (0.754, 0.888)	80.7 (80.4–81.0)	72.3 (61.4–83.2)	91.8 (84.2–99.5)
MRI inclusive criterion	0.752 (0.678, 0.827)	78.1 (77.8–78.4)	95.4 (90.3–100)	55.1 (41.2–69.0)

MRI strict criterion (favors specificity): SFO connected to the ligament is classified as NAF, while intersecting or not connected is classified as OSF; MRI inclusive criterion (favors sensitivity): SFO connected to or intersecting with the ligament is classified as NAF, while not connected is classified as OSF

### The correlation between MRI features and clinical symptoms

While SFO size demonstrated significant correlation with clinical symptoms, neither interposition of fluid-signal intensity nor SFO bone marrow edema showed significant associations (Table S3).

## Discussion

This study indicates that SFO predominantly affects younger individuals with unilateral predominance. NAF exhibited a male predominance (74/108, 68.5%), potentially attributable to greater physical activity exposure; OSF also showed a slight male preponderance. Laterality did not differ significantly (52 cases in the left, 62 right). Large-scale epidemiological studies on SFO remain unavailable. The typical clinical manifestations included chronic lateral malleolar pain (95/114, 83.3%) and joint instability (24/114, 21.1%), consistent with prior literature, the pathogenic mechanisms likely involve [2, 14, 17, 20], in NAF, the accessory lateral malleolar ligament fragment remains connected to the distal fibula via incomplete fibrous tissue, scar tissue, or synovial cavities. Variable tensile forces during activity increase fragment mobility, compromising joint stability; additionally, talofibular impingement involving the SFO and mechanical irritation of adjacent soft tissues can provoke synovitis and pain. Notably, pain was more frequent in the NAF. OSF-related pain may primarily stem from abnormal fragment mobility during high-intensity activity and secondary impingement syndromes.

In this study, lateral ankle instability (21.1%) was less frequent than pain (62.3%), which differs from the findings of Hasegawa et al [3], who reported a higher incidence of instability. This discrepancy likely reflects our stricter intraoperative criteria for diagnosing instability. We also observed a higher incidence of instability in NAF than in OSF, consistent with prior findings. The above results collectively demonstrate greater clinical necessity for surgical intervention versus conservative management in NAF patients.

This study revealed significantly higher incidences of irregular shape (78.5% vs. 21.5%) and rough margins (70.8% vs. 29.2%) in NAF compared to OSF, aligning with Han et al [14]. Pathologically, NAF essentially represents a fracture fragment undergoing asynchronous healing stages: early irregular woven bone and callus; intermediate replacement by lamellar bone with medullary fibrosis or fatty change; and late incomplete remodeling with cortical thickening or defects and re-established marrow with hematopoietic or normal fatty. This protracted heterogeneous process manifests radiographically as irregular morphology, cortical thickening or defects, narrowed medullary cavities, and a rough margin. Additionally, peripheral new bone may further accentuate irregularity and margin unevenness. Conversely, OSF is an independent accessory bone composed of regular lamellar bone and mature osteons, with intact cortical and cancellous bone, exhibiting radiologically regular shape, complete cortex, and smooth margin.

Nevertheless, occasional NAF cases may exhibit regular morphology and a smooth margin; we found this in 21.5% of the cases. Kwak et al observed progressive rounding in 11 of 17 ununited avulsion fractures [27]. Lee et al documented the transformation in 49 of 61 cases, correlating with initial fracture severity and healing duration [25]. Both NAF and OSF were predominantly medium to large in size (mean diameter: NAF 9.5 mm vs. OSF 8.8 mm, *p* > 0.05). Our measurements exceeded prior reports, attributable to methodological differences [1, 3, 14]: we used maximum 3D CT measurements, whereas earlier studies relied on lower-resolution, bidimensional radiographs. NAF demonstrated significantly higher mean, maximum, and minimum CT values than OSF. This likely reflects a greater cortical bone fraction in NAF (often predominantly cortical with little cancellous bone) and stage-dependent healing effects: callus formation and sclerosis elevate attenuation in early to mid-stage, whereas late-stage fatty or hematopoietic marrow replacement decreases it. Conversely, OSF exhibits lower attenuation due to normal marrow and fat content. However, long-standing NAF or OSF subjected to external forces or trauma may develop cystic degeneration and fibrosis, reducing CT values. Despite multifactorial CT value variability, NAF consistently shows higher attenuation than OSF.

Both NAF and OSF were most commonly located anteromedial to the lateral malleolus, without a significant difference. This predilection reflects biomechanical vulnerability: the ATFL is the weakest lateral collateral ligament of the ankle joint, accounting for 85% of ankle sprain; in addition, the distal fibula is thin and slender, while the calcaneus and talus are wide and thick, and their surface were covered with fibrocartilage that can cushion external force; as a result, avulsion fractures of the lateral malleolus most commonly occur at the distal fibula [1, 28]. Consequently, NAF primarily resides within the ATFL course anteromedial to the malleolus. Rare inferior or posteromedial occurrences may relate to PTFL injury or greater fragment mobility. While OSF may locate inferiorly, it never occurs posteromedially. NAF orientation was predominantly anteroposterior (89.2%), attributable to ATFL avulsion mechanics and proximal ligament traction, whereas the developmentally variant OSF exhibits variable orientation, though frequently anteroposterior.

The bony anatomical relationship of OSF to the lateral malleolus is mostly incompatible, whereas NAF more often appeared compatible, aligning with the findings of Han and Hasegawa et al [3, 14]. Hasegawa described these fractures as being positioned on an oblique plane, inclined medially from the superior to the inferior direction [3]. Han et al suggested that NAF can appear morphologically compatible with the inferior border of the malleolus [14]. The multiparameter logistic regression model (incorporating shape, margin, average, minimum, and maximum CT values, and bony anatomical relationship) outperformed any single parameter, with an accuracy of 74.3% and sensitivity of 91.3%, but a relatively low specificity of 56.8%. Among individual parameters, the maximum CT value showed the highest specificity of 81.1%.

Multiple studies have differentiated NAF from OSF on MRI by evaluating the SFO-ligament relationship: ligamentous attachment favors NAF, whereas the absence of direct attachment indicates OSF [18, 21–23]. Our data revealed ligamentous attachment as the predominant pattern in NAF (72.3%), contrasting with ligamentous non-connection or apposition in OSF (91.8%), with one case demonstrating PTFL attachment. A key limitation was our relatively thick MRI slices (4 mm), which, together with small fragment size, likely introduced partial volume effects and hampered accurate assessment of SFO-ligament interfaces [26]. Moreover, intraoperative or arthroscopic observations indicate that some OSFs may seem attached to the ATFL, but not at the ligament terminus, differing from the typical NAF attachment pattern. Current evidence suggests 3D-T2-weighted fast spin-echo sequences enhance SFO detection and ATFL relationship delineation [26], positioning thin-slice 3D protocols as pivotal for diagnostic accuracy improvement. Additionally, case reports have indicated that the Water Selective Cartilage Scan (3D_WATSc) sequence can aid OSF diagnosis by distinguishing cartilage from fibrous tissue, clearly depicting continuous cartilage surrounding the ossified focus, consistent with the mechanism of ossicle formation within the cartilaginous matrix of accessory bones [24]. In view of the limitations associated with conventional slice thickness, we recommend that future studies prioritize the use of advanced high-resolution MRI sequences such as 3D-FSE or WATSc imaging, which may further improve anatomical delineation and diagnostic accuracy.

MRI evidence of continuity between the SFO and the ligament strongly favored NAF, while discontinuity supported OSF, and apposition represented an overlapping manifestation. Using diagnostic criteria where continuity defines NAF and either discontinuity or apposition defines OSF yielded relatively high accuracy (80.7%) and specificity (91.8%), but lower sensitivity. Conversely, defining continuity or apposition as NAF and discontinuity as OSF significantly increased sensitivity (95.4%) but substantially reduced specificity. Overall, MRI demonstrated superior diagnostic performance to CT.

This study confirmed that the manifestation of lateral malleolar symptoms is primarily size-dependent, with medium and large SFOs being more likely to induce symptoms. aligning with Kim et al’s 9 mm threshold predicting poor outcomes with conservative treatment in chronic symptomatic SFOs [17]. Biomechanically, larger avulsion fragments imply more severe distal ligament injury and higher instability risk, greater displacement reflects increased ligament retraction with reduced inversion stability, and larger fragments are more prone to impingement. High PDWI signal in the SFO-fibula interval was common (63.2%) without intergroup differences, likely indicating synovitis and soft tissue impingement [14]. Gamble et al suggested the manifestation aids clinical decision-making, with its absence often correlating with a favorable response to conservative management, while its presence frequently necessitates surgical intervention [21]. Bone marrow edema was less frequent (22.8%) with no group differences. Impingement between the SFO and the talus or fibula can manifest as bone marrow edema in the affected regions. The relatively low detection rate of this finding in our cohort was likely attributable to two primary factors: the susceptibility of smaller fragments to underdiagnosis due to partial volume effects, and the sign is typically associated with acute or severe injury phases, whereas our study exclusively comprised chronic cases. Bone marrow edema and fluid cleft may reflect acute or evolving processes when observed in the setting of chronic injury. These features are not definitive predictors but could indicate recent aggravation, an acute-on-chronic injury state, or a healing phase.

Our study has several limitations. First, age is a significant influencing factor on bone density, with younger populations having higher content of hydroxyapatite and magnesium in their bone tissue, resulting in generally higher CT values [29]. Second, the sample size of asymptomatic patients is relatively small, which may affect the results of related statistical analyses. Third, arthroscopy has a limited field of view, and synovitis or inflammatory granulation can obscure structures, hindering confident assessment of the SFO-ligament relationship and potentially compromising classification accuracy [26, 27]. Lastly, selection bias may exist in the follow-up cohort: patients often cannot reliably recall prior ankle trauma, and baseline imaging data may not truly reflect the previous injury condition, so pre-existing ankle sprains with lateral malleolar avulsion fractures before baseline imaging cannot be excluded.

To the best of our knowledge, this study is the first to systematically differentiate between NAF and OSF using CT and MRI in a medium-sized sample. Our findings parallel earlier pediatric series [18, 21, 23], but significantly expand upon them with a larger, surgically verified adult cohort. MRI can visually display the anatomical relationship between the SFO and the lateral collateral ligaments of the ankle. Innovatively, two diagnostic criteria based on the ligament relationship seen in MRI were proposed and validated, showing superior diagnostic efficacy compared to CT. Nonetheless, CT provides more detailed anatomical morphological information. The combined application of both imaging modalities can offer a multidimensional basis for clinical decision-making.

## Supplementary information

Electronic Supplementary Material

## Data Availability

The dataset generated and analyzed during the study is available from the corresponding author upon reasonable request.

## Abbreviations

ATFL: Anterior talofibular ligament
AUC: Area under the receiver operating characteristic curve
CFL: Calcaneofibular ligament
NAF: Nonunited avulsion fracture
OSF: Os subfibulare
PTFL: Posterior talofibular ligament
ROC: Receiver operating characteristic curve
SFO: Subfibular ossicle
