# Supplementary material for: Enhanced subfibular ossicle diagnosis: CT-MRI integration of morphology and ligament attachments
Source: Insights Imaging. 2025 Nov 12;16:252. doi: 10.1186/s13244-025-02138-8 (PMC12612300; doi:10.1186/s13244-025-02138-8)
Supplement: Supplementary file 1 — Electronic Supplementary Material [file 13244_2025_2138_MOESM1_ESM.pdf]

Enhanced subfibular ossicle diagnosis: CT-MRI integration of morphology and ligament attachments

ELECTRONIC SUPPLEMENTARY MATERIAL

Table S1 Interobserver agreement for assessment of CT and MRI parameters in SFO

quantitative variables	*ICC* (95% CI)	*p* value	qualitative variables	*Kappa* (95% CI)	*p* value
average CT value	0.88 (0.82-0.93)	< 0.001	shape	0.91 (0.82-0.99)	< 0.001
minimum CT value	0.93 (0.88-0.97)	< 0.001	margin	0.91 (0.83-0.99)	< 0.001
maximum CT value	0.84 (0.75-0.91)	< 0.001	location	1.00 (1.00-1.00)	< 0.001
size	0.92 (0.85-0.96)	< 0.001	the orientation of the longest axis	0.90 (0.80-0.99)	< 0.001
			bony anatomical relationship	0.95 (0.89-1.00)	< 0.001
			ligament anatomical relationship	0.89 (0.82-0.96)	< 0.001

ICC, intraclass correlation coefficient.

Table S2 NAF versus OSF in CT findings

Characteristic	NAF<br>*n* = 65 (57%)[1]	OSF<br>*n* = 49 (43%)[1]	$t/Z/\chi 2^2$	p^2
**Shape**			15.221	< 0.001
irregular	51 (78.5%)	21 (42.9%)		
regular	14 (21.5%)	28 (57.1%)		
**Margin**			6.641	0.010
rough	46 (70.8%)	23 (46.9%)		
smooth	19 (29.2%)	26 (53.1%)		
**Size classification**				0.300
large	29 (44.6%)	16 (32.7%)		
medium	33 (50.8%)	28 (57.1%)		
small	3 (4.6%)	5 (10.2%)		
Size (mm)	9.5±2.6	8.8±2.6	1.344	0.182
**Average CT value (HU)**	484.5±164.4	392.5±157.4	2.459	0.016
**Minimum CT value (HU)**	140.6±74.7	113.5±76.2	2.005	0.045
**Maximum CT value (HU)**	791.7±243.5	661.1±257.0	2.244	0.028
**Location**				0.449
anteromedial	58 (89.2%)	47 (95.9%)		
inferior	5 (7.7%)	2 (4.1%)		
posteromedial	2 (3.1%)	0 (0.0%)		
**The orientation of the longest axis**				0.031
anteroposterior	58 (89.2%)	34 (69.4%)		
craniocaudal	4 (6.2%)	7 (14.3%)		
transverse	3 (4.6%)	8 (16.3%)		
**Bony anatomical relationship**			21.993	< 0.001
compatible	39 (60.0%)	8 (16.3%)		
noncompatible	26 (40.0%)	41 (83.7%)		

[1]n (%); Mean±SD; Median (Q1, Q3)

[2]Pearson's Chi-squared test; Fisher's Exact Test for Count Data; Two Sample t-test; Wilcoxon-Mann-Whitney Test

Table S3 The correlation between imaging features and clinical symptoms

Characteristic	Pain N = 71 (62%)[1]	Pain and instability N = 24 (21%)[1]	Asymptomatic N = 19 (17%)[1]	*t/Z/χ2*[2]	*p*[2]
**Size classification**				19.135	< 0.001
large	26 (36.6%)	15 (62.5%)	4 (21.1%)		
medium	42 (59.2%)	9 (37.5%)	10 (52.6%)		
small	3 (4.2%)	0 (0.0%)	5 (26.3%)		
**MRI-interposition of fluid signal intensity**				0.545	0.762
negative	22 (31.4%)	9 (39.1%)	7 (36.8%)		
positive	48 (68.6%)	14 (60.9%)	12 (63.2%)		
**MRI-bone marrow edema**				0.123	0.940
negative	53 (75.7%)	18 (78.3%)	15 (78.9%)		
positive	17 (24.3%)	5 (21.7%)	4 (21.1%)		

[1]n (%); Median (Q1, Q3); Mean±SD

[2]Pearson's Chi-squared test; Kruskal-Wallis rank sum test; One-way ANOVA
